# Crystal structure of *fac*-tri­chlorido­[tris­(pyridin-2-yl-*N*)amine]­chromium(III)

**DOI:** 10.1107/S2056989014027066

**Published:** 2015-01-01

**Authors:** Yukiko Yamaguchi-Terasaki, Takashi Fujihara, Akira Nagasawa, Sumio Kaizaki

**Affiliations:** a Toyama National College of Technology, Imizu Campus, 1-2 Ebie-neriya, Imizu city, Toyama 933-0293, Japan; b Comprehensive Analysis Center for Science, Saitama University, Shimo-Okubo 255, Sakura-ku, Saitama 338-8570, Japan; c Department of Chemistry, Graduate School of Science and Engineering, Saitama University, Shimo-Okubo 255, Sakura-ku, Saitama 338-8570, Japan; d Department of Chemistry, Graduate School of Science, Osaka University, Machikaneyama-cyo 1-1, Toyonaka, Osaka, Japan

**Keywords:** crystal structure, chloride ligand, pyridine ligand, chromium(III) complex, *facial* structural arrangement.

## Abstract

In the neutral complex mol­ecule of the title compound, *fac*-[CrCl_3_(tpa)] [tpa is tris­(pyridin-2-yl)amine; C_15_H_12_N_4_], the Cr^III^ ion is bonded to three N atoms that are constrained to a *facial* arrangement by the tpa ligand and by three chloride ligands, leading to a distorted octa­hedral coordination sphere. The average Cr—N and Cr—Cl bond lengths are 2.086 (5) and 2.296 (4) Å, respectively. The complex mol­ecule is located on a mirror plane. In the crystal, a combination of C—H⋯N and C—H⋯Cl hydrogen-bonding inter­actions connect the mol­ecules into a three-dimensional network.

## Abstract 2

The Cr^III^ ion in the title compound is bonded to three N atoms that are constrained to a *facial* arrangement by the tris­(pyridin-2-yl)amine ligand and by three chloride ligands, leading to a distorted octa­hedral coordination sphere.

## Chemical context   

One aspect of solvatochromism is the dependence of ligand-field parameters on the solvent coordination sphere. This has been demonstrated by measuring the ligand-field absorption spectra and/or multinuclear NMR spectra for several types of Cr^III^ complexes in previous studies (Kaizaki, 1996 ▸; Kaizaki & Takemoto, 1990 ▸; Terasaki & Kaizaki, 1995 ▸; Terasaki *et al.*, 1999 ▸; Yamaguchi-Terasaki *et al.*, 2007*a* ▸,*b* ▸,*c* ▸). As a part of the above-mentioned systematic investigations, we report here the crystal structure of the title compound, *fac*-[CrCl_3_(tpa)], (I), where tpa is tris­(pyridin-2-yl)amine.
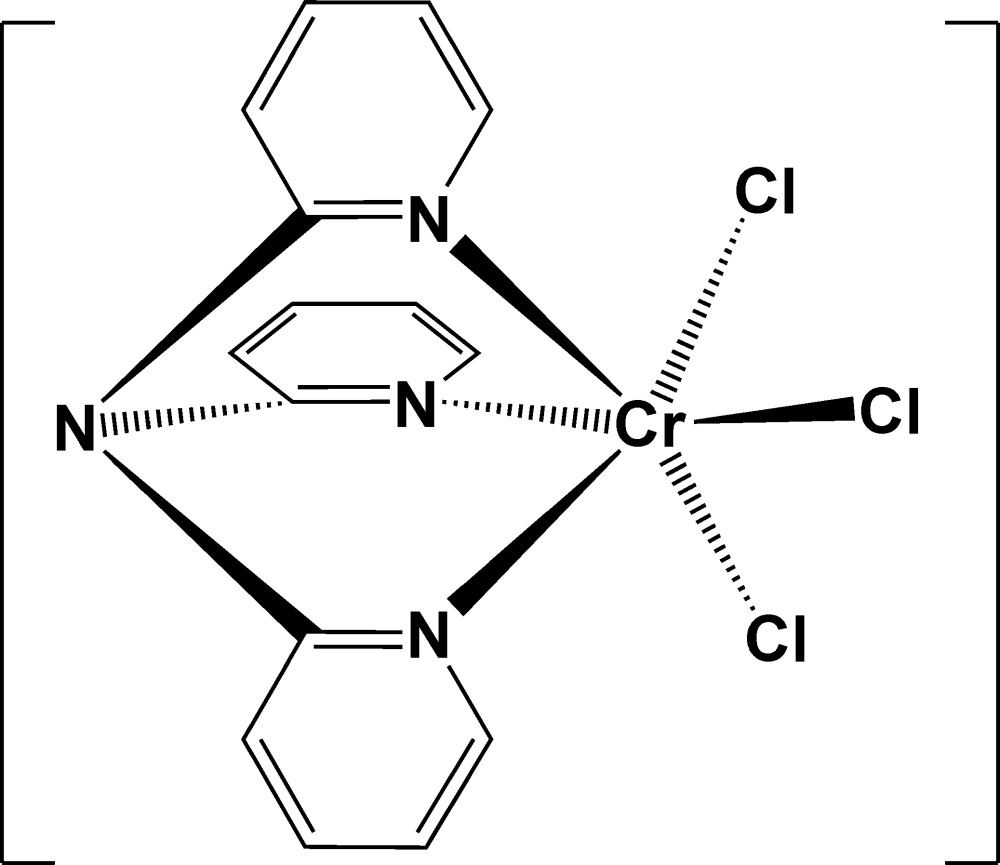


## Structural commentary   

**Figure 1:**
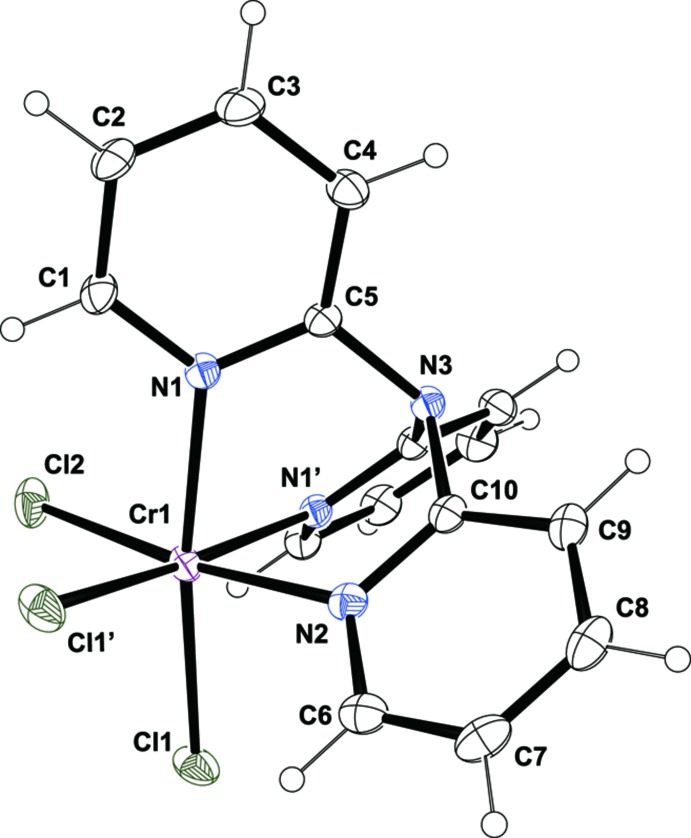
The mol­ecular structure of (I). Displacement ellipsoids are drawn at the 50% probability level. [Symmetry code: (′) *x*, −*y* + 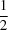, *z*.]

The mol­ecular structure of (I) is illustrated in Fig. 1 ▸. The Cr^III^ ion is coordinated by three N atoms that are constrained to a *facial* arrangement by the tpa ligand and three chloride ligands in a slightly distorted octa­hedral geometry. The entire complex mol­ecule is located on a mirror plane. The average Cr—N bond length of 2.086 (5) Å is comparable to that in the related tpa complex cation *fac*-[Cr(tpa)(H_2_O)_3_]^3+^ [2.040 (1) Å; Terasaki *et al.*, 2004 ▸]. In addition, the average Cr—Cl bond length of the coordinating chlorine atoms being in *trans* positions to the N atoms [2.296 (4) Å] is similar to those found for other pyridine-chromium(III) complexes, such as *mer*-[CrCl_3_(terpy)] [terpy is 2,2′,2′′-terpyridine; C_15_H_11_N_3_; 2.292 (1) Å] (Cloete *et al.*, 2007 ▸); *mer*-[CrCl_3_py_3_] [py is pyridine, C_5_H_5_N; 2.320 (7) Å] (Howard & Hardcastle, 1985 ▸) or *mer*-[CrCl_3_(Etpy)_3_] [Etpy is 4-ethyl­pyridine, C_7_H_9_N_3_; 2.320 (7) Å] (Modec *et al.*, 2000 ▸). All bond lengths and angles within the pyridine rings are within normal ranges. The dihedral angles between the least-squares planes of the pyridine rings are 58.33 (6) and 63.37 (8)°.

## Supra­molecular features   

**Figure 2:**
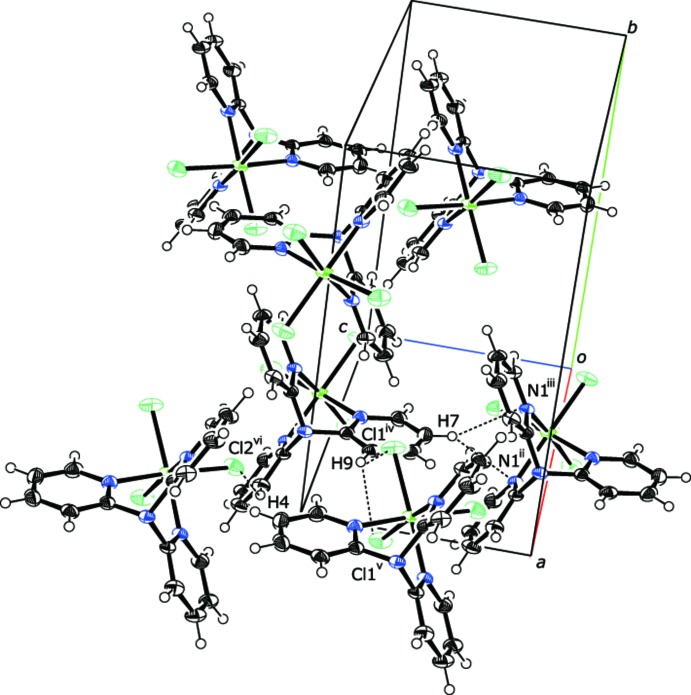
Hydrogen-bonding inter­actions in the crystal structure of (I), shown as black dashed lines. [Symmetry codes: (ii) *x*, −*y* + 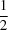, *z* − 1; (iii) *x*, *y*, *z* − 1; (iv) *x* + 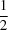, *y*, −*z* + 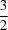; (v) *x* + 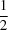, −*y* + 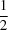, −*z* + 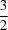; (vi) *x* + 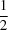, −*y* + 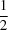, −*z* + 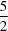.]

The chlorine atoms act as hydrogen-bond acceptors, forming inter­molecular C—H⋯Cl hydrogen bonds with the pyridine rings (Fig. 2 ▸, Table 1 ▸). In addition, C—H⋯N hydrogen-bonding inter­actions are also present, consolidating the mol­ecules into a three-dimensional network.

## Synthesis and crystallization   

*fac*-[CrCl_3_(tpa)] was synthesized according to a previously reported procedure (Kaizaki & Legg, 1994 ▸). Green crystals of (I) suitable for X-ray analysis were obtained by slow cooling from the reaction solution. UV–vis(DMSO): *λ*_max_(*∊*) = 720 (16), 645 (37), 464 (59) nm (L mol^−1^ cm^−1^). Elemental analysis, calculated for C_15_H_12_C_l3_CrN_4_: C, 44.31, H, 2.97, N, 13.78%; found: C, 44.29; H, 2.99; N, 13.76%.

## Refinement   

Crystal data, data collection and structure refinement details are summarized in Table 2 ▸. The H atoms were placed in calculated positions, with C—H = 0.95 Å, and refined using a riding model, with *U*_iso_(H) = 1.2*U*_eq_.

## Supplementary Material

Crystal structure: contains datablock(s) global, I. DOI: 10.1107/S2056989014027066/wm5095sup1.cif

Structure factors: contains datablock(s) I. DOI: 10.1107/S2056989014027066/wm5095Isup2.hkl

CCDC reference: 1038512

Additional supporting information:  crystallographic information; 3D view; checkCIF report

## Figures and Tables

**Table 1:** Hydrogen-bond geometry (, )

*D*H*A*	*D*H	H*A*	*D* *A*	*D*H*A*
C7H7N1^i^	0.95	2.75	3.578(5)	146
C7H7N1^ii^	0.95	2.75	3.578(5)	146
C9H9Cl1^iii^	0.95	2.82	3.447(4)	124
C9H9Cl1^iv^	0.95	2.82	3.447(4)	124
C4H4Cl2^v^	0.95	2.77	3.534(4)	138

Symmetry codes: (i) 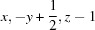; (ii) 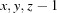; (iii) 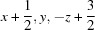; (iv) 
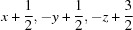
; (v) 
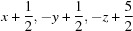
.

**Table 2:** Experimental details

Crystal data	
Chemical formula	[CrCl_3_(C_15_H_12_N_4_)]
*M*_r_	406.64
Crystal system, space group	Orthorhombic, *P* *n* *m* *a*
Temperature (K)	150
*a*, *b*, *c* ()	15.152(13), 13.704(12), 8.014(7)
*V* (^3^)	1664(2)
*Z*	4
Radiation type	Mo *K*
(mm^1^)	1.17
Crystal size (mm)	0.06 0.05 0.04

Data collection	
Diffractometer	Bruker APEXII CCD area detector
Absorption correction	Multi-scan (*SADABS*; Bruker, 2014 ▸)
No. of measured, independent and observed [*I* > 2(*I*)] reflections	15895, 1779, 1401
*R*_int_	0.061
(sin /)_max_ (^1^)	0.625

Refinement	
*R*[*F* ^2^ > 2(*F* ^2^)], *wR*(*F* ^2^), *S*	0.038, 0.113, 1.20
No. of reflections	1779
No. of parameters	118
H-atom treatment	H-atom parameters constrained
_max_, _min_ (e ^3^)	0.66, 0.51

Computer programs: *APEX2*, *SAINT* and *XPREP* (Bruker, 2014 ▸), *SHELXS2014*, *SHELXL2014* and *XCIF* (Sheldrick, 2008 ▸) and *ORTEP-3 for Windows* (Farrugia, 2012 ▸).

## supplementary crystallographic information

### Crystal data

**Table:**

[CrCl_3_(C_15_H_12_N_4_)]	*D*_x_ = 1.623 Mg m^−^^3^
*M**_r_* = 406.64	Mo *K*α radiation, λ = 0.71073 Å
Orthorhombic, *P**n**m**a*	Cell parameters from 3388 reflections
*a* = 15.152 (13) Å	θ = 2.7–26.4°
*b* = 13.704 (12) Å	µ = 1.17 mm^−^^1^
*c* = 8.014 (7) Å	*T* = 150 K
*V* = 1664 (2) Å^3^	Needle, green
*Z* = 4	0.06 × 0.05 × 0.04 mm
*F*(000) = 820	

### Data collection

**Table:**

Bruker APEXII CCD area-detector diffractometer	1779 independent reflections
Radiation source: Bruker TXS fine-focus rotating anode	1401 reflections with *I* > 2σ(*I*)
Bruker Helios multilayer confocal mirror monochromator	*R*_int_ = 0.061
Detector resolution: 8.333 pixels mm^-1^	θ_max_ = 26.4°, θ_min_ = 2.7°
φ and ω scans	*h* = −18→18
Absorption correction: multi-scan (*SADABS*; Bruker, 2014)	*k* = −17→17
*T*_min_ = ?, *T*_max_ = ?	*l* = −10→10
15895 measured reflections	

### Refinement

**Table:**

Refinement on *F*^2^	0 restraints
Least-squares matrix: full	Hydrogen site location: inferred from neighbouring sites
*R*[*F*^2^ > 2σ(*F*^2^)] = 0.038	H-atom parameters constrained
*wR*(*F*^2^) = 0.113	*w* = 1/[σ^2^(*F*_o_^2^) + (0.0593*P*)^2^ + 0.050*P*] where *P* = (*F*_o_^2^ + 2*F*_c_^2^)/3
*S* = 1.20	(Δ/σ)_max_ < 0.001
1779 reflections	Δρ_max_ = 0.66 e Å^−^^3^
118 parameters	Δρ_min_ = −0.51 e Å^−^^3^

### Special details

**Table:**

Geometry. All esds (except the esd in the dihedral angle between two l.s. planes) are estimated using the full covariance matrix. The cell esds are taken into account individually in the estimation of esds in distances, angles and torsion angles; correlations between esds in cell parameters are only used when they are defined by crystal symmetry. An approximate (isotropic) treatment of cell esds is used for estimating esds involving l.s. planes. Least-squares planes (x,y,z in crystal coordinates) and deviations from them (* indicates atom used to define plane) - 0.0000 (0.0001) x + 13.7040 (0.0118) y - 0.0000 (0.0000) z = 3.4260 (0.0030) * 0.0000 (0.0000) N2 * 0.0000 (0.0000) C6 * 0.0000 (0.0000) C7 * 0.0000 (0.0000) C8 * 0.0000 (0.0000) C9 * 0.0000 (0.0000) C10 Rms deviation of fitted atoms = 0.0000 - 0.2014 (0.0170) x - 7.1958 (0.0145) y + 6.8195 (0.0076) z = 4.9979 (0.0213) Angle to previous plane (with approximate esd) = 58.326 ( 0.080 ) * -0.0024 (0.0017) N1_$6 * -0.0057 (0.0019) C1_$6 * 0.0081 (0.0020) C2_$6 * -0.0029 (0.0020) C3_$6 * -0.0049 (0.0019) C4_$6 * 0.0078 (0.0018) C5_$6 Rms deviation of fitted atoms = 0.0057 0.2014 (0.0171) x + 7.1958 (0.0145) y + 6.8195 (0.0076) z = 8.8847 (0.0184) Angle to previous plane (with approximate esd) = 63.371 ( 0.081 ) * -0.0024 (0.0017) N1 * -0.0057 (0.0019) C1 * 0.0081 (0.0020) C2 * -0.0029 (0.0020) C3 * -0.0049 (0.0019) C4 * 0.0078 (0.0018) C5 Rms deviation of fitted atoms = 0.0057

### Fractional atomic coordinates and isotropic or equivalent isotropic displacement parameters (Å^2^)

**Table:**

	*x*	*y*	*z*	*U*_iso_*/*U*_eq_	
C1	0.89047 (17)	0.06834 (18)	1.2036 (3)	0.0287 (6)	
H1	0.8290	0.0568	1.2158	0.034*	
C2	0.94935 (19)	0.0033 (2)	1.2726 (4)	0.0336 (7)	
H2	0.9287	−0.0516	1.3336	0.040*	
C3	1.0382 (2)	0.0186 (2)	1.2521 (4)	0.0355 (7)	
H3	1.0796	−0.0262	1.2972	0.043*	
C4	1.06674 (17)	0.0997 (2)	1.1654 (4)	0.0309 (6)	
H4	1.1279	0.1117	1.1495	0.037*	
C5	1.00429 (16)	0.16273 (18)	1.1026 (3)	0.0224 (6)	
C10	1.0039 (2)	0.2500	0.8449 (5)	0.0239 (8)	
C6	0.8905 (3)	0.2500	0.6541 (5)	0.0328 (9)	
H6	0.8292	0.2500	0.6295	0.039*	
C7	0.9499 (3)	0.2500	0.5256 (5)	0.0378 (10)	
H7	0.9295	0.2500	0.4136	0.045*	
C8	1.0383 (3)	0.2500	0.5577 (5)	0.0358 (10)	
H8	1.0797	0.2500	0.4688	0.043*	
C9	1.0667 (2)	0.2500	0.7224 (5)	0.0293 (9)	
H9	1.1277	0.2500	0.7490	0.035*	
Cl1	0.75326 (4)	0.37578 (5)	0.89695 (10)	0.0388 (2)	
Cl2	0.75220 (6)	0.2500	1.26071 (14)	0.0379 (3)	
Cr1	0.83104 (4)	0.2500	1.01679 (7)	0.0239 (2)	
N1	0.91747 (13)	0.14774 (15)	1.1195 (3)	0.0225 (5)	
N2	0.91684 (19)	0.2500	0.8144 (4)	0.0256 (7)	
N3	1.03099 (19)	0.2500	1.0163 (4)	0.0227 (7)	

### Atomic displacement parameters (Å^2^)

**Table:**

	*U*^11^	*U*^22^	*U*^33^	*U*^12^	*U*^13^	*U*^23^
C1	0.0299 (13)	0.0202 (13)	0.0360 (16)	−0.0023 (11)	0.0030 (12)	0.0013 (12)
C2	0.0433 (16)	0.0167 (13)	0.0407 (17)	0.0001 (12)	0.0019 (13)	0.0046 (12)
C3	0.0413 (16)	0.0190 (14)	0.0462 (18)	0.0045 (12)	−0.0051 (13)	0.0039 (13)
C4	0.0268 (13)	0.0264 (14)	0.0394 (16)	0.0011 (11)	−0.0038 (12)	−0.0018 (13)
C5	0.0259 (13)	0.0145 (13)	0.0267 (14)	0.0022 (10)	−0.0001 (10)	−0.0007 (10)
C10	0.0296 (19)	0.0151 (17)	0.027 (2)	0.000	0.0023 (16)	0.000
C6	0.039 (2)	0.024 (2)	0.035 (2)	0.000	−0.0078 (19)	0.000
C7	0.061 (3)	0.027 (2)	0.026 (2)	0.000	0.000 (2)	0.000
C8	0.052 (3)	0.022 (2)	0.034 (2)	0.000	0.015 (2)	0.000
C9	0.034 (2)	0.0163 (18)	0.038 (2)	0.000	0.0086 (18)	0.000
Cl1	0.0309 (4)	0.0240 (4)	0.0615 (5)	0.0046 (3)	−0.0127 (3)	0.0036 (3)
Cl2	0.0295 (5)	0.0309 (6)	0.0534 (7)	0.000	0.0160 (4)	0.000
Cr1	0.0191 (3)	0.0170 (3)	0.0356 (4)	0.000	−0.0005 (2)	0.000
N1	0.0240 (11)	0.0161 (10)	0.0276 (12)	−0.0001 (8)	0.0011 (9)	−0.0018 (9)
N2	0.0288 (16)	0.0196 (16)	0.0285 (17)	0.000	−0.0012 (13)	0.000
N3	0.0225 (15)	0.0162 (15)	0.0295 (17)	0.000	0.0011 (12)	0.000

### Geometric parameters (Å, º)

**Table:**

C1—N1	1.344 (3)	C6—C7	1.367 (6)
C1—C2	1.377 (4)	C6—H6	0.9500
C1—H1	0.9500	C7—C8	1.363 (7)
C2—C3	1.372 (4)	C7—H7	0.9500
C2—H2	0.9500	C8—C9	1.388 (6)
C3—C4	1.380 (4)	C8—H8	0.9500
C3—H3	0.9500	C9—H9	0.9500
C4—C5	1.376 (4)	Cl1—Cr1	2.2983 (15)
C4—H4	0.9500	Cl2—Cr1	2.2909 (19)
C5—N1	1.338 (3)	Cr1—N2	2.079 (3)
C5—N3	1.440 (3)	Cr1—N1^i^	2.087 (2)
C10—N2	1.342 (4)	Cr1—N1	2.087 (2)
C10—C9	1.366 (5)	Cr1—Cl1^i^	2.2983 (15)
C10—N3	1.434 (5)	N3—C5^i^	1.440 (3)
C6—N2	1.345 (5)		
			
N1—C1—C2	121.9 (3)	C10—C9—C8	117.9 (4)
N1—C1—H1	119.1	C10—C9—H9	121.1
C2—C1—H1	119.1	C8—C9—H9	121.1
C3—C2—C1	119.2 (3)	N2—Cr1—N1^i^	85.14 (10)
C3—C2—H2	120.4	N2—Cr1—N1	85.14 (10)
C1—C2—H2	120.4	N1^i^—Cr1—N1	84.35 (13)
C2—C3—C4	119.4 (3)	N2—Cr1—Cl2	172.72 (9)
C2—C3—H3	120.3	N1^i^—Cr1—Cl2	89.46 (8)
C4—C3—H3	120.3	N1—Cr1—Cl2	89.46 (8)
C5—C4—C3	118.3 (3)	N2—Cr1—Cl1^i^	89.69 (8)
C5—C4—H4	120.9	N1^i^—Cr1—Cl1^i^	171.91 (6)
C3—C4—H4	120.9	N1—Cr1—Cl1^i^	89.03 (8)
N1—C5—C4	122.9 (2)	Cl2—Cr1—Cl1^i^	95.12 (5)
N1—C5—N3	116.9 (2)	N2—Cr1—Cl1	89.69 (8)
C4—C5—N3	120.2 (2)	N1^i^—Cr1—Cl1	89.03 (8)
N2—C10—C9	123.6 (4)	N1—Cr1—Cl1	171.91 (6)
N2—C10—N3	117.1 (3)	Cl2—Cr1—Cl1	95.12 (5)
C9—C10—N3	119.3 (3)	Cl1^i^—Cr1—Cl1	97.18 (7)
N2—C6—C7	121.6 (4)	C5—N1—C1	118.3 (2)
N2—C6—H6	119.2	C5—N1—Cr1	118.28 (17)
C7—C6—H6	119.2	C1—N1—Cr1	123.39 (18)
C8—C7—C6	120.3 (4)	C10—N2—C6	117.7 (3)
C8—C7—H7	119.8	C10—N2—Cr1	118.2 (2)
C6—C7—H7	119.8	C6—N2—Cr1	124.0 (3)
C7—C8—C9	118.9 (4)	C10—N3—C5	112.33 (18)
C7—C8—H8	120.5	C10—N3—C5^i^	112.33 (18)
C9—C8—H8	120.5	C5—N3—C5^i^	112.4 (3)
			
N1—C1—C2—C3	−1.4 (4)	C2—C1—N1—Cr1	−178.7 (2)
C1—C2—C3—C4	1.1 (4)	C9—C10—N2—C6	0.000 (1)
C2—C3—C4—C5	0.2 (4)	N3—C10—N2—C6	180.000 (1)
C3—C4—C5—N1	−1.2 (4)	C9—C10—N2—Cr1	180.000 (1)
C3—C4—C5—N3	177.9 (2)	N3—C10—N2—Cr1	0.000 (1)
N2—C6—C7—C8	0.000 (1)	C7—C6—N2—C10	0.000 (1)
C6—C7—C8—C9	0.000 (1)	C7—C6—N2—Cr1	180.000 (1)
N2—C10—C9—C8	0.000 (1)	N2—C10—N3—C5	63.9 (2)
N3—C10—C9—C8	180.000 (1)	C9—C10—N3—C5	−116.1 (2)
C7—C8—C9—C10	0.000 (1)	N2—C10—N3—C5^i^	−63.9 (2)
C4—C5—N1—C1	1.0 (4)	C9—C10—N3—C5^i^	116.1 (2)
N3—C5—N1—C1	−178.1 (2)	N1—C5—N3—C10	−64.4 (3)
C4—C5—N1—Cr1	−179.9 (2)	C4—C5—N3—C10	116.4 (3)
N3—C5—N1—Cr1	1.0 (3)	N1—C5—N3—C5^i^	63.4 (4)
C2—C1—N1—C5	0.3 (4)	C4—C5—N3—C5^i^	−115.8 (3)

Symmetry code: (i) *x*, −*y*+1/2, *z*.

### Hydrogen-bond geometry (Å, º)

**Table:**

*D*—H···*A*	*D*—H	H···*A*	*D*···*A*	*D*—H···*A*
C7—H7···N1^ii^	0.95	2.75	3.578 (5)	146
C7—H7···N1^iii^	0.95	2.75	3.578 (5)	146
C9—H9···Cl1^iv^	0.95	2.82	3.447 (4)	124
C9—H9···Cl1^v^	0.95	2.82	3.447 (4)	124
C4—H4···Cl2^vi^	0.95	2.77	3.534 (4)	138

Symmetry codes: (ii) *x*, −*y*+1/2, *z*−1; (iii) *x*, *y*, *z*−1; (iv) *x*+1/2, *y*, −*z*+3/2; (v) *x*+1/2, −*y*+1/2, −*z*+3/2; (vi) *x*+1/2, −*y*+1/2, −*z*+5/2.
